# Development of a Rat Model for Glioma-Related Epilepsy

**DOI:** 10.3390/ijms21196999

**Published:** 2020-09-23

**Authors:** Charlotte Bouckaert, Charlotte Germonpré, Jeroen Verhoeven, Seon-Ah Chong, Lucas Jacquin, Georges Mairet-Coello, Véronique Marie André, Karine Leclercq, Christian Vanhove, Filip De Vos, Caroline Van den Broecke, Ingeborg Goethals, Benedicte Descamps, Sam Donche, Evelien Carrette, Wytse Wadman, Paul Boon, Kristl Vonck, Robrecht Raedt

**Affiliations:** 1 4Brain, Department of Head and Skin, Ghent University, 9000 Ghent, East Flanders, Belgium; Charlotte.Bouckaert@UGent.be (C.B.); Charlotte.Germonpre@UGent.be (C.G.); Evelien.Carrette@UGent.be (E.C.); Wytse.Wadman@UGent.be (W.W.); Paul.Boon@UZGent.be (P.B.); Kristl.Vonck@UGent.be (K.V.); 2 Department of Pharmaceutical Analysis, Ghent University, 9000 Ghent, East Flanders, Belgium; Jeroen.Verhoeven@UGent.be (J.V.); FilipX.DeVos@UGent.be (F.D.V.); cvdbroec@me.com (C.V.d.B.); 3 Early Solutions, Neuroscience Therapeutic Area, UCB Pharma, 1420 Braine-l’Alleud, Brabant Wallon, Belgium; Seon-Ah.Chong@ucb.com (S.-A.C.); Lucas.Jacquin@ucb.com (L.J.); Georges.Mairet-Coello@ucb.com (G.M.-C.); VeroniqueMarie.Andre@ucb.com (V.M.A.); Karine.Leclercq@ucb.com (K.L.); 4 Department of Electronics and information systems, Ghent University Hospital, 9000 Ghent, East Flanders, Belgium; Christian.Vanhove@UGent.be (C.V.); Benedicte.Descamps@UGent.be (B.D.); 5 Department of Diagnostic Sciences, Ghent University Hospital, 9000 Ghent, East Flanders, Belgium; Ingeborg.Goethals@UGent.be (I.G.); Sam.Donche@UGent.be (S.D.)

**Keywords:** glioma, seizures, high-grade glioma rat model, glioma-related epilepsy, video-EEG monitoring

## Abstract

Seizures are common in patients with high-grade gliomas (30–60%) and approximately 15–30% of glioblastoma (GB) patients develop drug-resistant epilepsy. Reliable animal models are needed to develop adequate treatments for glioma-related epilepsy. Therefore, fifteen rats were inoculated with F98 GB cells (GB group) and four rats with vehicle only (control group) in the right entorhinal cortex. MRI was performed to visualize tumor presence. A subset of seven GB and two control rats were implanted with recording electrodes to determine the occurrence of epileptic seizures with video-EEG recording over multiple days. In a subset of rats, tumor size and expression of tumor markers were investigated with histology or mRNA in situ hybridization. Tumors were visible on MRI six days post-inoculation. Time-dependent changes in tumor morphology and size were visible on MRI. Epileptic seizures were detected in all GB rats monitored with video-EEG. Twenty-one days after inoculation, rats were euthanized based on signs of discomfort and pain. This study describes, for the first time, reproducible tumor growth and spontaneous seizures upon inoculation of F98 cells in the rat entorhinal cortex. The development of this new model of GB-related epilepsy may be valuable to design new therapies against tumor growth and associated epileptic seizures.

## 1. Introduction

Glial tumors or gliomas represent 80% of all malignant brain tumors [1,2]. According to the World Health Organization (WHO) classification system, gliomas can be histologically classified as low-grade gliomas (LGG; ±15% of all gliomas) or high-grade gliomas (HGG; ±85% of all gliomas). Eighty percent of HGG are grade IV gliomas, also termed glioblastoma (GB), representing the most common and malignant brain tumor and carrying a poor prognosis, with medium survival rates of 12–17 months after diagnosis [3,4,5,6,7].

Seizures represent a common symptom in GB and occur in 30–60% of patients with GB [3,8]. Brain tumor-related seizures typically have a focal onset and can be associated with alteration of consciousness and secondary generalization [3]. Up to 30% of patients with GB suffer from drug-resistant epilepsy (DRE), resulting in significant morbidity and negative impact on quality of life [3,9,10,11].

Investigating the mechanisms of GB development in animal models is essential to develop effective treatments. Animal models provide the opportunity to study the characteristics of tumor development and the occurrence of GB-related seizures as well as the pathogenic processes underlying epileptogenesis. This may lead to the identification of new targets for treatment development against GB invasion, GB-related seizures, and ultimately, help to improve quality of life in patients with GB.

Different studies investigated the mechanisms of epileptogenesis in brain tumors, but these mechanisms remain poorly understood and are likely to be multifactorial, involving both host and tumor factors [12]. In vitro analysis on brain slices in rat and mouse models of GB showed neuronal hyperexcitability in peritumoral regions (1–2 mm from main tumor mass), which triggers epileptiform activity [13,14]. This phenomenon was also observed in a clinical setting, where magnetoencephalography (MEG) demonstrated abnormal low frequency activity in the peritumoral area in 13 out of 20 patients with tumor-related epilepsy [15]. Pathophysiological mechanisms that contribute to glioma-associated neuronal hyperexcitability include ischemia, neuronal network disruption/reorganization, altered gap junction coupling, abnormal ion and neurotransmitter levels, neural plasticity, and pronounced gliosis with chronic inflammatory changes [9,16,17,18,19,20]. Recently, it has been suggested that bidirectional interactions between neurons and glioma play an important role in glioma progression: neuronal activity increases glioma growth and gliomas increase neuronal activity [18,19].

Although many rodent glioma models have been described [21], these models were not designed to study the evolution of GB over time in relation to the occurrence of epilepsy and certainly not with personalized video-EEG monitoring. In this paper, we describe the progression and histopathological properties as well as the occurrence of seizures and the development of epilepsy in the syngeneic Fischer/F98 rat model of GB.

## 2. Results

### 2.1. Tumor Growth

**Figure 1:**
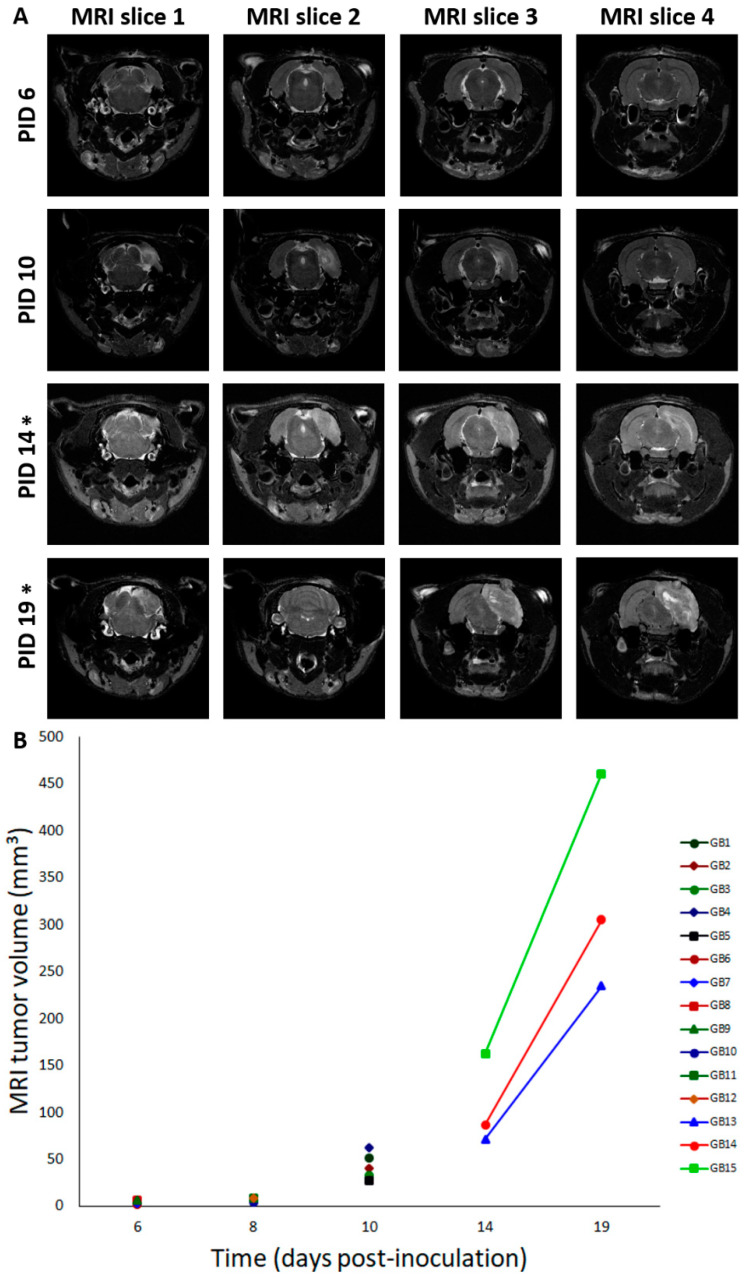
Tumor evolution and deformation of the brain over time. (**A**) Deformation of the brain over time. This figure shows consecutive (MRI slice 1 to 4, posterior to anterior) T2 MRI images (slice thickness: 0.6 mm—interslice thickness: 1.2 mm) in a rat brain at different time points: PID6: Hyperintense T2 signal at inoculation site (GB9); PID10: three distinct tumor zones can be identified (GB5); PID14: large tumor starts to deform the brain (GB14); PID19: massive tumor deforms the brain, pushes on the brainstem, and causes a midline shift (GB14). T2 MRI images “MRI slice 2” are taken around the inoculation site, while T2 MRI images “MRI slice 4” are taken close to the electrodes implanted in the hippocampi. PID—post-inoculation day. * indicates that images of PID14 and PID19 are obtained in the same GB animal (GB14). (**B**) Tumor volumes on MRI for different GB rats in function of time. In this graph, MRI tumor volumes for individual rats are shown for different points in time (days post-inoculation). Note that overlay of rats is present on PID6 (GB6–7 and GB8–9) and PID8 (GB11–12). Lines connect MRI tumor volumes of the same rats at different time points.

Fifteen male F344/IcoCrl rats were inoculated with 20,000 F98 cells in 5 µL phosphate-buffered saline (PBS) in the right entorhinal cortex (GB1–15). In four control animals (C1–4), 5 µL of PBS was injected at the same location. We refer to the day of inoculation as post-inoculation day 0 (PID0). MRI was performed in GB rats at different time points after inoculating F98 glioma cells in the entorhinal cortex. It has to be noted that MRI is performed in different rats at the different time points, except at PID3 and PID10, and at PID14 and PID19 (Table 1, M). At all time points, except at PID3, presence of a tumor was demonstrated with MRI. Representative T2 images at different time points post-inoculation are shown in Figure 1A. MRI tumor volumes in GB rats at different time points post-inoculation can be found in Figure 1B. The size of the tumor increased with time post-inoculation. In all four GB rats scanned on PID6 (GB6–9), a hyperintense T2 signal was observed at the inoculation site, indicating the presence of a tumor (median 4.5 mm^3^, range 1.4–6.7 mm^3^). On PID8, a larger homogeneous hyperintense zone (median 8.8 mm^3^, range 4.2–8.9 mm^3^) was observed at the inoculation site in all three scanned GB rats (GB10–12). On PID10, heterogeneous tumors (median 40.6 mm^3^, range 27.5–62.1 mm^3^) with three distinct tumor zones could be identified in all five scanned GB rats (GB1–5) on T2 images: (1) a central zone of hyperintense signal; (2) a rim of a relatively hypointense signal; and (3) an outer rim of less intense signal (Figure 1A, MRI slice 2 at PID10). In the three GB rats scanned on PID14 and PID19 (GB13–15), tumors were large (median 86.9 mm^3^, range 71.2–162.6 mm^3^) and started to deform the brain on PID14 and became massive (median 304.8 mm^3^, range 234.8–460.6 mm^3^), pushing on the left hemisphere and brainstem on PID19. MRI was only performed in two control animals on PID8 (C3–4). In these animals, no abnormalities were observed.

### 2.2. Seizures

After confirmation of tumor growth on the MRI, recording electrodes were implanted in both hippocampi and in the ipsilateral parietal cortex in a subset of GB rats (GB1–9). Two out of four control rats (C1–2) were also implanted with electrodes. Time points of electrode implantation varied between PID5 and PID12 (Table 1, E). GB1 died during implantation surgery. In GB3, EEG recordings could not be interpreted due to bad signal quality. The remaining nine animals (seven GB rats and two control rats) were monitored continuously with video-EEG starting immediately after electrode implantation until euthanasia (Table 1). The number of seizures per day per rat, categorized per stage, is shown in Table 2.

**Figure 2:**
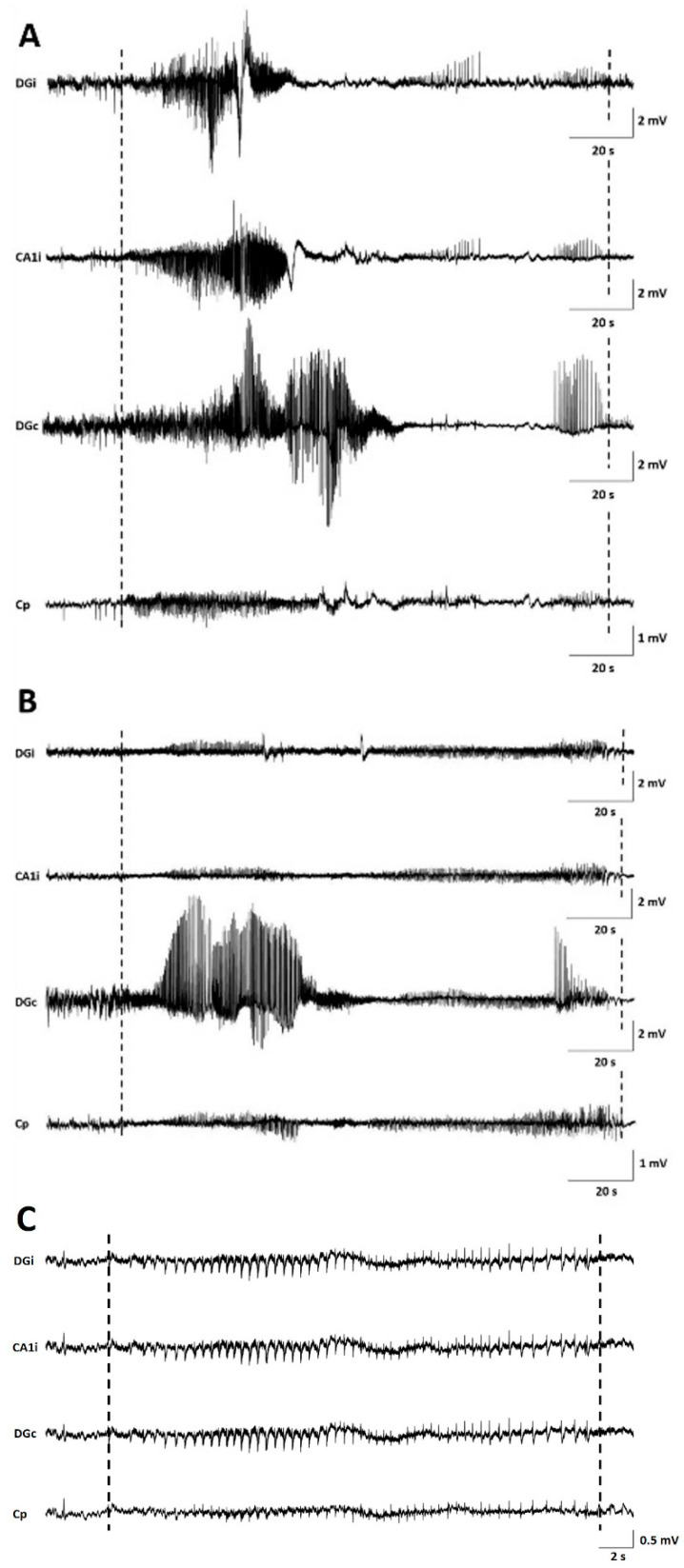
Examples of seizures on EEG. (**A**) representative spontaneous stage 1 seizure at PID15 in GB5. (**B**) representative spontaneous stage 1 seizure at PID20 in GB5. (**C**) representative spontaneous stage 5 seizure at PID6 in C1. Dashed lines indicate beginning and end of the seizures. The first channel represents EEG recorded from the ipsilateral dentate gyrus (DGi), the second channel EEG recorded from the ipsilateral CA1 (CA1i), the third channel EEG recorded from the contralateral dentate gyrus (DGc), and the fourth channel EEG recorded from the surface electrode at the tumor border (Cp).

Seizures occurred in all monitored GB rats between PID8 and PID21 with a high interindividual variability in the number of seizures and day of occurrence. Overall, the number of seizures per GB rat did not increase over time nor the fraction of rats having seizures. No significant difference in seizure occurrence during the light phase (median 11, range 5–18) versus dark phase (median 4, range 0–37) was found (Wilcoxon Signed Rank Test, *p* = 0.993). Median severity of seizures in GB rats was score 1 on the modified Racine Scale (range 1–5). GB rats had significantly more (Wilcoxon test, *p* = 0.018) non-convulsive (median 10, range 6–31) than convulsive seizures (median 6, range 0–21). Median electrographic seizure duration in GB rats was 75.4 s (range 24–257 s). The electrographic duration of non-convulsive seizures (median 60.4 s, range 24–203.5 s) was significantly shorter (Mann–Whitney U Test, *p* = 0.004) compared to convulsive seizures (median 109.69 s, range 24.5–257 s). In both monitored control rats, stage 5 seizures were observed, but these seizures were limited to day 1 and 2 after electrode implantation (PID6 and 7). These convulsive stage 5 seizures showed different EEG and behavioral characteristics than the majority of stage 5 seizures in GB rats, although GB rats implanted with electrodes on PID7–8 did show similar seizures shortly after electrode implantation. These specific type of stage 5 seizures (indicated with * in Table 2) were primary generalized, with simultaneous electrographic onset on all four EEG channels (Figure 2C) and rats showed immediate convulsive behavior at electrographic seizure onset and not the typical sequential evolution in behavior (initial freezing and head nodding followed by forelimb clonus and later on, hindlimb clonus and falling). Furthermore, these stage 5 seizures (median 47.5 s, range 24.5–85 s) were significant shorter (Mann–Whitney U Test, *p* < 0.001) in duration than other stage 5 seizures (median 132.7 s, range 58.5–257 s).

In GB rats, a clear attenuation of the EEG signal amplitude with time post-inoculation was observed first in ipsilateral but finally also in the contralateral hippocampus. Electrographic seizures could still be clearly discriminated (Figure 2).

### 2.3. Expression of Proliferative and Glial Markers in the Tumor and Its Microenvironment

**Figure 3:**
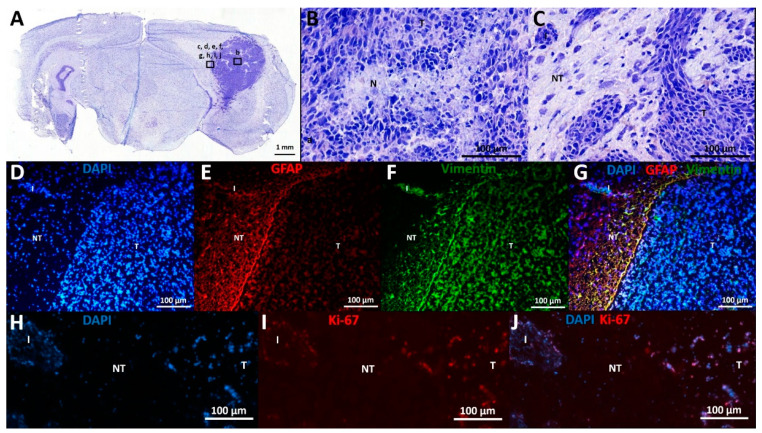
Histological and immunohistochemical analysis. (**A**–**C**) Cresyl violet staining: (**A**) This overview picture shows a tumor-containing coronal slice stained with cresyl violet. Tumor tissue can be clearly identified on the right of the slice, appearing dark purple as opposed to less intense stained normal brain tissue. (**B**) Cresyl violet staining in tumor tissue, 20× magnification: This figure shows a necrotic spot surrounded by palisading tumor cells (N), as well as nuclear atypia and marked hypercellularity. (**C**) Cresyl violet staining at tumor border, 20× magnification: This figure shows the transition from tumor (T) to non-tumor (NT) tissue, including an infiltrative spot (I). (**D**–**G**) Expression of GFAP and vimentin: (**D**) DAPI staining: Tumor tissue contains more densely packed cells compared to neighboring non-tumor tissue, as demonstrated by DAPI positive nuclei. (**E**) GFAP staining: GFAP labeling is mainly found at tumor border and diffuse in tumor tissue. (**F**) Vimentin staining: Vimentin labeling is found at tumor border and diffuse in tumor tissue. (**G**) DAPI, vimentin, and GFAP overlay: Many double positive cells align at tumor border. (**H**–**J**) Expression of Ki-67: (**H**) DAPI staining. (**I**): Ki-67 staining. (**J**): DAPI and Ki-67 overlay: Ki-67 positive cells are seen in tumor tissue and infiltrative spots, whereas most cells in adjacent non-tumor tissue are Ki-67 negative. T—tumor tissue; NT—non-tumor tissue; I—infiltrative spot; N—necrotic spot.

Histological characteristics of F98 GB tumors at PID21 included necrotic areas surrounded by tumor cells, marked hypercellularity, nuclear atypia, and infiltrative growth (Figure 3A–C).

Immunohistochemical analysis confirmed the expression of astrocytic lineage markers (GFAP, vimentin) by the GB cells (Figure 3D–G). Vimentin and GFAP labeling are both dense at the tumor border, where these cells display a palisade arrangement in their cell processes (Figure 3E–F). However, there is high heterogeneity in GFAP expression throughout the tumor, ranging from 0 to 100% GFAP^+^ cells per evaluated random high power field (of 40×-magnification), with a higher number of GFAP^+^ cells further away from the tumor core, whereas vimentin expression remains high (approximately 100%) in all tumor regions. In tumor tissue, 46 ± 3% (mean ± SD) of cells were Ki-67 positive, confirming their mitotic state. Only 2 ± 2% (mean ± SD) of cells in the adjacent normal brain parenchyma were found to be Ki-67 positive (Figure 3H–J).

**Figure 4:**
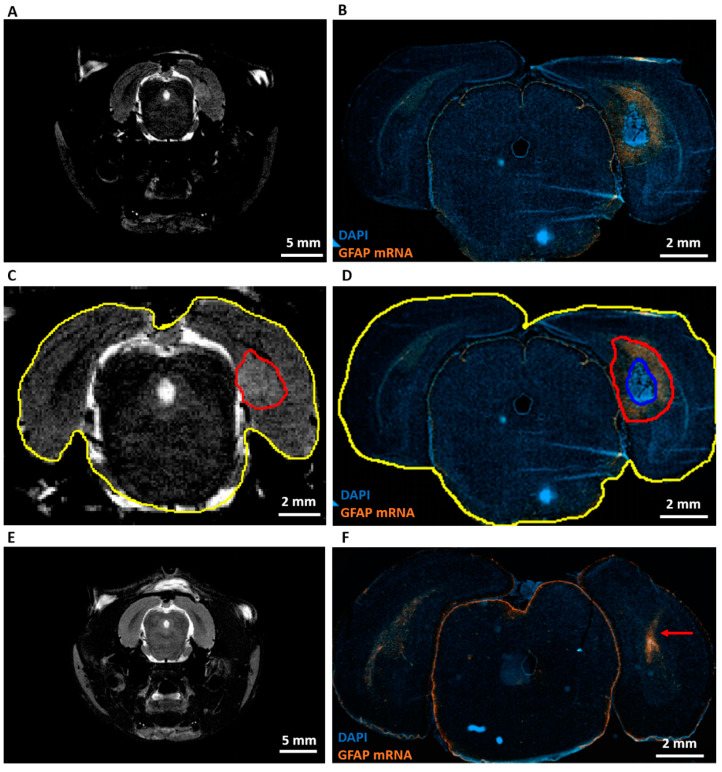
Expression of GFAP mRNA and comparison with MRI. (**A**) Representative T2 MRI image of a GB rat on PID8. A homogeneous hyperintense zone can be identified ipsilateral of the inoculation site. (**B**) Representative RNAscope image of the same GB rat on PID9. This image shows a large tumor (strong DAPI staining) surrounded by high level of GFAP mRNA expression. (**C**) Alignment of the hyperintense zone (red) and whole brain (yellow) on the T2 MRI image of a GB rat on PID8. Relative size of the hyperintense zone is calculated by dividing its area by the total area of the brain slice on MRI. (**D**) Alignment of tumor (blue) and tumor + rim of strong GFAP mRNA expression (red). Relative areas are calculated by dividing the blue and red area by the total area of the brain slice on the RNA scope image (yellow). (**E**) Representative T2 MRI image of a control rat (C4) on PID8. (**F**) Representative RNAscope image of C4 on PID9. The red arrow indicates the injection tract, in which there is an increase in GFAP mRNA expression.

RNA scope analysis at PID9 (Table 1, R) showed high levels of GFAP mRNA around the area of dense tumor cells in GB rats (Figure 4B). However, almost no GFAP mRNA was found in the tumor, whereas heterogeneous GFAP expression was observed inside the tumor by immunohistochemistry at PID21. When MRI images at PID8 (Figure 4A) were compared with RNAscope images at PID9, the hyperintense signal on the MRI (Figure 4C, aligned in red) extended beyond the area of dense tumor cells (Figure 4D, aligned in blue) and included a large part of a GFAP^+^ astrogliotic rim around the dense tumor core as well (Figure 4D, aligned in red). Hyperintense areas on MRI images were two to three times larger than areas of dense tumor cells on RNAscope slices. These hyperintense areas were 27 ± 5% (mean ± SD) smaller than the composite areas including the dense tumor cells and astrocytic rim on RNAscope slices (Table 3). GFAP mRNA levels were increased in control rats along the injection tract (Figure 4F).

### 2.4. End-Stage Tumor Volumes

Tumor volumes at PID21 of GB6, GB7, GB8, and GB9 were 243, 262, 151, and 145 mm^3^. These respective animals had a total number of 52, 40, 9, and 22 seizures. Based on this low number of animals, no clear link between total number of seizures and end-stage tumor volumes was seen.

Tumor volumes (expressed as means ± SEM) evaluated on T2 MRI at PID19 (333 ± 66 mm^3^) tended to be larger (*p* = 0.229) than histological tumor volumes at PID21 (200 ± 30 mm^3^).

## 3. Discussion

This study characterizes tumor development at various time points using both structural imaging and histology following F98 GB inoculation in the entorhinal cortex. In addition, the study describes, for the first time, the epileptic phenotype associated with tumor evolution in this model.

Tumor inoculation in the right entorhinal cortex was successful in all animals as all animals developed tumor lesions. On day 6 post-inoculation, GB tumors could be demonstrated with preclinical MRI at the site of inoculation. Tumor development was very similar to that of human GB [6]. Lesions in our model progressed in size over time and invaded the brain. Necrotic zones in GB tumors were detected in animals on MRI at PID10, 14, and 19. At PID14, MRI shows a clear shift forward of the right hippocampus as a consequence of the presence of the space-occupying lesion. At PID19, MRI showed massive tumors causing a real deformation of the brain, including the left-brain hemisphere. This deformation of the brain could possibly explain the attenuation of the EEG signal resulting from loss of input from the entorhinal cortex. Ultimately, tumor evolution led to the decision of euthanasia of the animals based on signs of serious discomfort predicting nearing mortality. The trend towards larger tumor volumes calculated from MRI images compared to tumor volumes calculated from histological slices was expected as T2 MRI visualizes the tumor itself, as well as the infiltrative border of the tumor and brain edema [22]. RNAscope results showed that GB tumors are surrounded by reactive gliosis reflected by the high level of GFAP mRNA surrounding the dense tumor core. Comparison with MRI images of PID8 confirmed that this reactive gliosis was also hyperintense on T2 MRI. Immunohistochemistry for GFAP and vimentin at PID21 confirmed the glial origin of the developed tumors. Vimentin was profuse in tumor tissue and at the tumor border, whereas GFAP staining was only profuse at the tumor border and more heterogeneously expressed throughout the tumor. At PID9, high levels of GFAP mRNA were also detected in tissue surrounding the tumor, but not in tumor tissue. Vimentin expression is an early event in normal glial differentiation, while GFAP only appears in later stages [23,24]. Therefore, a possible explanation is that the majority of tumor cells in these GB tumors were neoplastic astrocytes in a dedifferentiated state. Dense GFAP labeling in the brain parenchyma surrounding the tumor reflects the presence of reactive astrocytes [24]. Tumor-associated astrocytes can be activated by neighboring glioma cells and reactive astrocytes promote proliferation, invasion, and treatment resistance of GB via a complex interaction (involving gap junctions, ion channels and transporters, chemokines, and cytokines) with the tumor microenvironment [25]. Many cells in the GB tumor tissue stained positive for the proliferative marker Ki-67 and thus, actively divided [26], confirming the high grade of invasiveness. We found a Ki-67 index of 47.30%, which is very comparable to the proliferative index of 46% reported by Belloli et al. [27] in a similar F98 GB rat model.

All GB rats demonstrated seizures throughout the video-EEG monitoring period (PID8–21), with the majority of seizures being non-convulsive. Primary generalized convulsive seizures occurred in SHAM-inoculated control rats (implanted with electrodes on PID5) and in GB rats implanted with electrodes on PID7 and PID8 but not on PID11 and PID12. The short interval between two consecutive brain surgeries (inoculation—electrode implantation) might have stimulated the occurrence of these post-implantation seizures. Acute inflammatory response due to the injection procedure may have had a negative influence on the outcome of the electrode implantation surgery. Our RNAscope analysis for GFAP expression in control animals indeed shows an inflammatory response along the injection tract at PID8. The occurrence of seizures in the first days after electrode implantation and their absence at later time points in control rats injected with saline in brain parenchyma is fully in line with an earlier study performed by our lab [28]. Similar acute seizures post-surgery in sham-operated experimental controls have also been reported by Andrade et al. [29].

In GB rats, we found no clear correlation between number of seizures/day and time post-inoculation, arguing against a tumor-related mass effect as the sole cause of seizures. One other study by Köhling et al. investigated epileptic activity in a GB rat model [13]. All transplanted rats (*n* = 4) showed short epileptiform discharges on EEG lasting several seconds, but in contrast to our observations, no epileptic seizures with behavioral abnormalities (judged on the basis of actograms and by visual inspection). Possible explanations for the lack of seizures in their model could be different rat strain (Wistar vs. Fischer), transplantation site (somatosensory vs. entorhinal cortex), and cell type (C6 cells vs. F98 cells). Indeed, C6 cells show circumscribed growth patterns, whereas F98 cells are infiltrative [30]. Apart from tumor type (seizures are more common in LGG than in HGG), tumor location also influences the risk for epilepsy [8]. The incidence of epilepsy is higher for cortical than for non-cortical tumors, regardless of the involved lobe [9]. Entorhinal, piriform, and perirhinal cortex are highly interconnected with each other and with other limbic structures, and are, therefore, particularly prone to the genesis and propagation of ictal discharges [31]. Tumors involving mesiotemporal and insular (paralimbic) structures frequently result in medication-resistant epilepsy [9].

Mouse models for GB-related epilepsy are described in two studies by Buckingham et al. In the first study, SCID mice were either implanted with human GBM12/GBM22 xenografts (2.5 × 10^5^ cells/10 µL) or U251GFP human glioma cell line (5 × 10^5^ cells/10 µL) in the left hemisphere. Very short epileptiform EEG events were seen in 37% of the mice, mostly associated with a subtle behavioral phenotype [14]. In the second study by Buckingham et al., GBM22 xenografts (1–1.5 × 10^5^ cells) were implanted into one or both hemispheres. Seizures were detected in 80% of the mice [32]. Both methodologies have not led to a standardized mouse model protocol for GB-related epilepsy as there is no clear description of the exact injection site and electrode positions, different xenografts were used, and a high variability in seizure occurrence (37% vs. 80%) and seizure duration (0.5 s vs. 26 s) was reported in both studies. In another study, rat-derived glioma C6 cells (1 × 10^5^ cells/5 µL) were inoculated into the right motor cortex of adult nude mice. Video-EEG was recorded 24/7 for 3 weeks and spontaneous seizures were measured in glioma-inoculated mice with an increase in seizure duration over time, whereas no seizures were measured in sham-implanted mice. However, the main focus of that study was not to establish an in vivo model for glioma-related epilepsy, but to investigate a role for glioma-induced alterations in KCC2 in the peritumoral region in tumor-associated epilepsy and therefore, no detailed characterization of seizures was given [33].

Previously mentioned models were not designed to study the evolution of GB over time in relation to the occurrence of epilepsy and certainly not with personalized video-EEG monitoring. Therefore, to our knowledge, we are the first to describe progression and histopathological properties along with the occurrence of seizures and characteristics of these seizures in the syngeneic Fischer/F98 rat model of GB.

This study had some limitations. First, a limited number of animals—seven GB rats and two control rats—were monitored with video-EEG. Despite this limitation, we did find a clear difference between groups in the occurrence of seizures with all GB rats, but none of the SHAM rats, displaying seizures in the later stages of video-EEG monitoring. Therefore, the occurrence of glioma-related seizures in this model is consistent. Second, we injected only one type of glioma cell. To rule out that observed seizures are not cell line-specific, the results need to be confirmed in a model using another glioma cell line. Third, because we did not use MRI-compatible electrodes, it was not possible to combine MRI with video-EEG monitoring, which will be needed to directly link tumor characteristics (e.g., size, spread, edema) to tumor-related seizures. Fourth, this study showed reactive gliosis around the GB, but inflammatory responses were not addressed in detail. More extensive tissue analysis for markers of reactive microglia (e.g., Iba-1), axonal injury (e.g., NFL), and T-lymphocytes (e.g., CD45) should be performed to further characterize the inflammatory response. A multimodal approach combining video-EEG, MRI, and tissue analysis in this model will make it straightforward to screen treatments for both tumor growth and tumor-related seizures.

## 4. Materials and Methods

### 4.1. F98 Cells

F98 (ATTC) rat GB cells were cultured as monolayers in Dulbecco’s Modified Eagle Medium (DMEM), supplemented with 10% fetal calf serum (FCS), 1% penicillin–streptomycin, 1% l-glutamine, and 0.1% fungizone (all products for cell culture were purchased from Invitrogen^®^ (Merelbeke, Belgium)) and placed in an incubator at 37 °C and 5% CO_2_.

### 4.2. Animals

Nineteen male F344/IcoCrl rats (Charles River^®^) with ages of 9–10 weeks and mean body weight of 245 ± 22 g were used. The study was approved by the animal ethics committee of the Faculty of Medicine and Health Sciences of Ghent University (ECD 17/111). All animals were kept and handled according to European guidelines (Directive 2010/63/EU) and housed under environmentally controlled conditions: 12 h light/dark cycle, temperature between 21 and 24 °C, and humidity between 55% and 65%. Food and water were provided ad libitum.

### 4.3. Inoculation

All animals were anesthetized with a mixture of medical oxygen and isoflurane (induction: 5%; maintenance: 2%) and immobilized in a stereotaxic frame. Body temperature was maintained at 37 °C by a thermoregulated heating pad. The glioma cell suspension was stereotactically injected in the right entorhinal cortex (anterioposterial (AP): −8.0 mm and mediolateral (ML): +4.5 mm relative to bregma, dorsoventral (DV): −4.1mm relative to dura) using an insulin syringe (BD 0.5 mL Insulin Syringe Microfine 0.33 mm (29G) × 12.7 mm) mounted on a pump for automatic injection (Stoelting Quintessential Stereotaxic Injector, Stoelting Co., Wood Dale, IL, USA). In 15/19 animals (GB1–15), 20,000 F98 cells in 5 µL phosphate-buffered saline (PBS) were injected over a period of 10 min. In four control animals (C1–4), 5 µL of PBS was injected. Of these control rats, two rats (C1–2) were used for video-EEG monitoring and two for RNAscope analysis preceded by MRI (Table 1). The syringe was kept in place for five minutes post-inoculation and then, slowly removed. The skin was sutured, and meloxicam was administered subcutaneously (1 mg/kg, 2 mg/mL) to manage pain due to surgery. In the remainder of this manuscript, we refer to the inoculation day as post-inoculation day 0 (PID 0).

### 4.4. Brain Imaging Using Micro-MRI Confirmation and Follow-Up of Tumor Growth

In vivo 7-tesla micro-MRI (PharmaScan 70/16, Bruker BioSpin, Ettlingen, Germany) was used to visualize brain lesions following inoculation to evaluate tumor growth (Table 1, M). Time points of MRI differed in subsets of rats. In the first group (GB1–5), MRI scans were performed on post-inoculation day (PID) 3. As no tumors could be visualized yet, MRI was repeated on PID10 to confirm tumor growth prior to electrode implantation on PID11 or 12. Since tumors were extensive on PID10, a second group of rats (GB6–9) were scanned on PID6 and implanted with electrodes on PID7 or 8, which allowed the initiation of video-EEG monitoring earlier. A third group of rats (GB10–12 and C3–4) were scanned on PID8 before perfusion on day 9 for RNAscope analysis (see below). In a fourth group of rats (GB13–15), MRI was performed on PID14 and 19 to visualize tumors at later stages, which is not feasible once electrodes are implanted for video-EEG monitoring.

Animals were anesthetized with a mixture of oxygen and isoflurane (induction: 5%; maintenance: 2%) and placed in the scanner, where the isoflurane-oxygen mixture was administered through a nose cone fixed onto the animal holder. A heating pad using a warm water circuit to maintain body temperature at 37 °C was placed underneath the animal. A volume coil (Rapid Biomedical, Rimpar, Germany) was placed around the head. Respiratory rate was monitored during the entire protocol. A localizer scan was performed, followed by a T2-weighted spin-echo scan (TR/TE 3661/37.1 ms, 109 μm isotropic in-plane resolution, slice thickness 0.6 mm, 4 averages, TA 9′45″).

MRI tumor volumes were defined as the extent of a hyperintense signal on MRI reflecting the tumor itself, the infiltrative border, and brain edema. These volumes were calculated from T2-weighted images using ImageJ (National Institutes of Health, Bethesda, MD, USA). On each MRI slice with visible lesions, the hyperintense signal around the inoculation site in the right-sided temporal lobe was marked and the surface area was determined. MRI tumor volumes per rat brain were calculated by summating the surface areas and by multiplying the mean surface area by 0.6 mm (0.6 mm slice thickness).

Moreover, qualitative aspects of MRI images were examined as well, such as deformation of the brain and MRI tumor characteristics over time.

### 4.5. Electrode Implantation for EEG Recording

One or two days after confirmation of tumor presence with MRI, a subset of animals (8/15 GB rats, GB2–9) were anesthetized with a mixture of medical oxygen (rate: 1 L/min) and isoflurane (induction: 5%; maintenance: 2%) and their heads were fixated in a stereotaxic frame. For recording of epidural EEG, a scalp electrode was placed over the right parietal cortex near the tumor border. Epidural scalp electrodes were custom-made and consisted of a stainless-steel micro screw (0.8 mm diameter, Plastics One, Roanoke, VA, USA) soldered to an insulated copper wire. At the connector side, electrode wires were sandwiched between two Winslow connector pins (267–7400, RS components, Colby, UK). Two additional scalp electrodes (serving as ground and spare ground) and two anchor screws were implanted in the os frontale, one anchor screw in the os parietale, and three anchor screws in the os occipitale. Two small craniotomies were made for placement of bipolar depth electrodes (tips 0.9 mm apart) in left and right hippocampus (AP: −5.0 mm; ML: +/−3.5 mm relative to bregma). Via a small incision in the dura, electrodes were inserted in the brain using audio control (i.e., unit activity). When the deepest contact point reached the dentate gyrus (−3.2 ± 0.6 mm below dura), electrodes were fixed to the skull and neighboring anchor screws using UV cement (Filtek Supreme XTE Plus Flowable 2 × 2 gr, 3M, Diegem, Belgium). At the end of the surgery, the connector pins of all electrodes were assembled in a connector block (female side exposed) and secured to the skull using metabond (Super-Bond C&B, Sunmedical, Shiga, Japan) and dental cement (Simplex Rapid fluid/powder, Kemdent, Swindon, UK). Meloxicam (1 mg/kg, 2 mg/mL) was administered subcutaneously after surgery. In two control rats, electrodes were implanted 5 days after injection of PBS (Table 1, E).

### 4.6. Continuous Video-EEG Monitoring

Continuous video-EEG monitoring was performed as described previously by Germonpré et al. and is explained here in short [28]. After electrode implantation surgery, animals were housed in neurophysiology cages (Plexiglas 29 × 29 × 45 cm) where they were connected to a video-EEG monitoring setup.

This setup consisted of a headstage carrying a 4-channel unity gain amplifier, a tether, and a 12-channel commutator (Plastics One, Roanoke, VA, USA), allowing the animals to move freely. EEG signals were high-pass filtered at 0.15 Hz and amplified 512×. A NiDAQ card (USB-6259, National Instruments device, Austin, TX, USA) with 32 analog input channels (8 rats, 4 channels/rat) was used to digitize the EEG at a sampling rate of 2 kHz (16-bit resolution, +/− 10 V input range).

The behavior of each animal was individually monitored with a camera (Raspberry Pi 3) in front of the cage. Both EEG and video were recorded continuously after electrode implantation until euthanasia. Rear and side walls were covered with mirror foil, making it possible to visualize the animal from all possible angles. Infrared lights allowed for visualization of the animal in the dark.

A MATLAB-based application was used to control the acquisition and storage of the EEG and video files.

Electrographic seizures were defined as rhythmic patterns of EEG spikes with evolving complexity and an amplitude exceeding at least three times the baseline amplitude and lasting more than 10 s. The start and end of seizures were determined using a subjective “by eye” evaluation of the EEG signal recorded from the ipsilateral dentate gyrus (Figure 2, DGi). If a seizure was detected on the EEG, corresponding videos were used to detect any clinical symptoms or to exclude non-epileptic activity (scratching, drinking, etc.). A modified Racine Behavioral Scale [34] was used to score behavioral changes during seizures: Stage 1: mouth and facial movements (incl. wet dog shakes), stage 2: head nodding, stage 3: unilateral forelimb clonus, stage 4: bilateral forelimb clonus and rearing, and stage 5: clonus of all limbs and falling. Based on this Racine scale, seizures were classified as non-convulsive (no clear motor component; stage 1–2) and convulsive (clear motor component; stage 3–5) seizures. The number of seizures per rat per stage was evaluated, as well as the number of GB/control rats with seizures per day and the number of (non-convulsive) seizures in GB/control rats with seizures per day. The duration of seizures, occurrence of seizures during light/dark phase, and median severity of seizures were also investigated.

### 4.7. Histology

All seven GB rats and both control rats that were monitored with video-EEG were euthanized at day 21 with an overdose of sodium pentobarbital (200 mg/kg, Vétoquinol S.A./N.V., Aartselaar, Belgium) which was administered intraperitoneally under isoflurane anesthesia. GB2–5 were transcardially perfused with PBS followed by 4% paraformaldehyde (PFA) (Table 1, H(●)). We found that PFA-based fixation of brain tissue, followed by sucrose-based cryoprotection and frozen section procedure, was unsuitable for histological analysis of tumor size as well as for immunohistochemical analysis in this model. Making free-floating sections with a thickness of 40 µm of these PFA-perfused brains resulted in tumor tissue detachment from the remaining brain tissue that led to teared slices without tumor tissue. This problem was solved by snap freezing fresh brain tissue and making 5-µm-thick frozen sections that were immediately mounted. Therefore, GB6–9 and C1–2 were transcardially perfused with saline at room temperature followed by ice cold PBS (Table 1, H). Brains were isolated and immediately frozen in liquid nitrogen.

Coronal sections (5 µm) were prepared using a cryostat (Leica, Wetzlar, Germany) from these latter six brains. Each 100th slice was mounted directly on a glass slide and a cresyl violet staining was performed. Some additional slices for immunohistochemical analysis were also mounted and stored in a −80 °C freezer (see Section 4.8. Immunohistochemistry). Tumor volumes on PID21 were calculated based on digitized histological slices using ImageJ (National Institutes of Health, Bethesda, MD, USA). On each slice, the tumor border (i.e., border aligning darker stained areas and significant infiltration zones) was marked and surface area was determined. Histological tumor volumes per rat brain were calculated by summating tumor surface areas determined on all tumor containing slices and multiplying this average surface area by 0.5 mm (5 µm slice thickness + 495 µm interslice thickness). Furthermore, histological characteristics such as necrosis, cell morphology, and infiltration were investigated on these digitized slices.

### 4.8. Immunohistochemistry

Immunohistochemical stainings for the astrocytic markers glial fibrillary acidic protein (GFAP) and vimentin, and for the cell proliferation marker Ki-67 were performed. Briefly, 5 µm-mounted slices were first treated with 0.5% and 1% H_2_O_2_ in PBS for 30 and 60 min, respectively, to block endogenous peroxidase activity. Secondly, sections were incubated for 45 min with a blocking solution containing PBS, 0.2% Triton X-100 and 0.4% Fish Skin Gelatin. Next, primary antibodies were applied for 1 h at room temperature at the following dilutions: rabbit anti-GFAP (1:1000; DAKO, Heverlee, Belgium), mouse anti-vimentin (1:250; DAKO, Heverlee, Belgium), and anti-rabbit Ki-67 (1:100; Fisher Scientific, Merelbeke, Belgium). After extensive washing, the sections were incubated for 1 h with goat anti-rabbit-Alexa fluor 594 (1:1000; DAKO, Heverlee, Belgium) and/or goat anti-mouse-Alexa fluor 488 (1:1000; DAKO, Heverlee, Belgium) secondary antibodies. After thorough rinsing, DAPI (1 µg/mL) was applied for 1 min for nuclear staining. A droplet of Vectashield Mounting Medium Fluorescence was applied to the slices and slices were coverslipped. For Ki-67 staining, five random high-power fields (40×) in tumor tissue and five in non-tumor tissue were evaluated by counting positive and negative nuclei. The estimated number of positive Ki-67+ nuclei was divided by the estimated number of counted nuclei (DAPI+) to obtain an estimated proliferation index. GFAP and vimentin expression in tumor and non-tumor tissue was also estimated by counting positive and negative nuclei in these high-power fields.

### 4.9. RNA Scope Assay

RNAscope was used in order to visualize GFAP mRNA in GB-containing brain slices. This is a novel RNA in situ hybridization (ISH) technology with a unique probe design strategy that allows simultaneous signal amplification and background suppression to achieve single-molecule visualization with the preservation of tissue morphology [35]. GB10–12 and C3–4 were sacrificed 9 days post-inoculation and their brains were collected in RNAse-free conditions, followed by snap freezing in liquid nitrogen (Table 1, R). Brains were then sliced to provide 12-µm-thick coronal sections using a microtome and kept at −80 °C until further processing.

GFAP mRNA was targeted with RNAscope^®^ Probe-Rn-Gfap-C2 reagent (ACDbio, Newark, CA), a mixture of 20 probes targeting the region from 1539 to 2534bp of the rat GFAP mRNA sequence (accession number: NM_017009.2).

Sections were fixed for 1 h in 4% PFA at 4 °C directly out of the −80 °C freezer and rinsed three times in PBS before dehydration. The dehydration procedure was done at room temperature. Sections were incubated in 50% EtOH, 70% EtOH, and 100% EtOH for 5 min each and in 100% EtOH for 10 min. The sections were air-dried for 5 min before to draw a barrier around it with a hydrophobic barrier pen. The barrier was left to dry for 1–2 min and RNAscope protease IV (ACDbio, Newark, CA, USA) diluted at 1/15 in PBS was added to cover the sections for 30 min at room temperature. The protease was rinsed two times for 2 min in PBS and the GFAP probe was added to cover the sections. The sections were inserted in the HybEZ Oven (ACDbio, Newark, CA, USA) for 2 h at 40 °C in the HybEZ Humidity control tray (ACDbio, Newark, CA, USA) with a wet HybEZ Humidifying paper (ACDbio, Newark, CA, USA). The 50× RNAscope^®^ Probe-Rn-Gfap-C2 was prepared at 1× with probe diluent and warmed for 10 min at 40 °C before use. After probe hybridization, the samples were rinsed two times for 2 min in wash buffer (ACDbio, Newark, CA, USA) and kept overnight in Saline Sodium Citrate (0.75 M NaCl in 75 mM sodium citrate (pH 7.0)) buffer.

For the amplification and detection steps, reagents were brought to room temperature before the start. RNAscope Multiplex FL V2 AMP1 (ACDbio, Newark, CA, USA), the preamplifier, hybridizing only pairs of probes to limit the risk of unspecific binding, was added to the section for 30 min at 40 °C. Then, RNAscope Multiplex FL V2 AMP2 (ACDbio, Newark, CA, USA), the amplifier, hybridizing the preamplifier to provide a support for the label probe and RNAscope Multiplex FL V2 AMP3 (ACDbio, Newark, CA, USA) serving as linker for the preamplifier and amplifier, were added for 30 and 15 min respectively at 40 °C.

For the detection step, we used RNAscope Multiplex FL V2 HRP-C2 (ACDbio, Newark, CA, USA), binding to the amplifier of the C2 channel, and added it for 15 min at 40 °C. The TSA Plus Cyanine 3 System (PerkinElmer, Waltham, MA, USA) revealing the C2 channel and RNAscope Multiplex FL V2 HRP blocker (ACDbio, Newark, CA, USA) blocking HRP from further reaction with the TSA system were added for 30 and 15 min, respectively, at 40 °C.

For the amplification and detection steps, two rinses for 2 min with wash buffer at room temperature after each step were performed.

Finally, the sections were stained for 45 s with RNAscope Multiplex FL V2 DAPI (ACDbio, Newark, CA, USA) and mounted with antifade mounting medium.

Images were acquired with the Axio Scan.Z1 (Zeiss, Oberkochen, Germany) with 40× objective and analyzed with Zen microscope software (Zeiss, Oberkochen, Germany).

Per GB rat, one slice near the inoculation site was selected and the area of dense tumor cells (marked by strong DAPI signal) and the composite area of dense tumor cells + astrogliotic rim surrounding the dense tumor cells (marked by high levels of GFAP mRNA) were determined. These areas were compared with the area of hyperintense MRI signal at the inoculation site detected in the corresponding animals on PID8. For comparison, the areas were normalized by dividing them by the total brain area on the selected RNAscope/MRI slices, respectively.

### 4.10. Statistical Analysis

All statistical analyses were performed in SPSS version 26 (Chicago, IL, USA) with the level of statistical significance set at 0.05. Unless otherwise stated, data are expressed as medians and range. Non-parametric tests were used due to the low number of animals. Mann–Whitney U Test was used to compare the duration of non-convulsive and convulsive seizures in GB rats as well as to compare MRI tumor volumes and histological tumor volumes. Wilcoxon Signed Rank Test was used to compare the occurrence of seizures during light vs. dark phase and to compare the number of convulsive vs. non-convulsive seizures in GB rats.

## 5. Conclusions

Until today, no standardized in vivo rodent model for GB-related epilepsy exists. The development of this rat model for GB-related epilepsy allows for the monitoring of seizures in a reproducible manner and the characterization of the tumor microenvironment in epileptic rats. This will allow screening for new therapies against tumor growth and associated epileptic seizures. Since survival is only 21 days in this model, we suggest initiating treatments as early as possible. Furthermore, to investigate treatments against GB-associated seizures, sufficient animals need to be included in the experiments as a high interindividual variability in the number and occurrence of seizures was seen.

## Figures and Tables

**Table 1:**
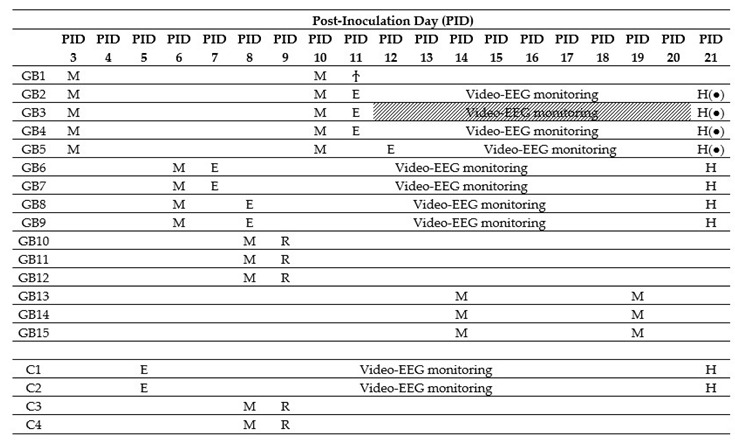
Overview of timing of the different procedures per animal. MRI (M) was performed in different animals at different time points. After confirmation of tumor growth, electrodes were implanted (E) in a subset of animals. GB1 died during electrode implantation (Ϯ). GB3 was left out for analysis due to bad EEG signals (shaded). Tissue from monitored animals was collected on PID21 for histological analysis (H). Tissue from GB10–12 and C3–4 was collected the day after MRI for RNAscope analysis (R).

M—MRI scan; E—EEG electrode implantation; R—RNAscope analysis; H—histological analysis; H(●)—no histological analysis due to problems with tissue processing.

**Table 2:**
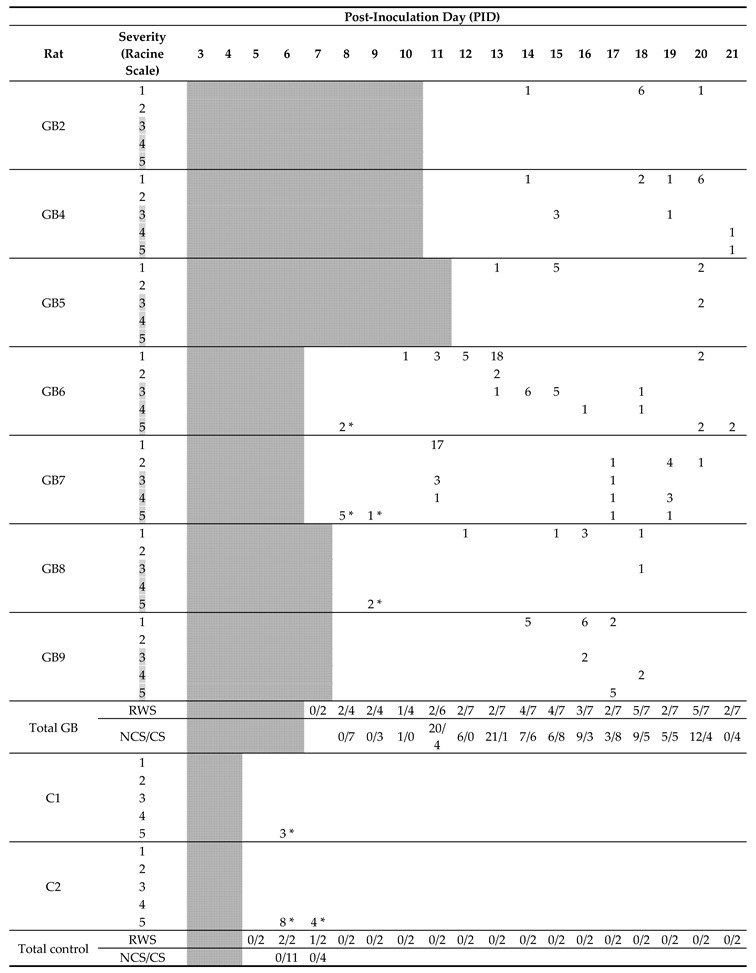
Timeline with occurrence of seizures in individually monitored rats. Number of seizures per day and per stage during the entire video-EEG monitoring period in individual rats is indicated in the table. As rats were implanted with electrodes at different time points, days before electrode implantation—on which no video-EEG monitoring was performed—are shaded. Furthermore, fraction of rats with seizures per day (RWS) and total number of non-convulsive seizures (NCS) and convulsive seizures (CS) are indicated. Based on the Racine scale, stage 1 and 2 seizures with no clear motor component were classified as non-convulsive seizures and stage 3–5 seizures with a clear motor component were classified as convulsive seizures. Convulsive seizure stages are shaded in grey. Primary generalized seizures shortly after electrode implantations are marked with *.

**Table 3:** MRI vs. RNAscope. This table summarizes the different relative areas calculated on MRI and RNAscope images. We can see that hyperintense areas on MRI extend beyond strongly DAPI-stained areas on RNAscope, but are smaller than areas including a high level of GFAP mRNA on RNAscope.

	MRI PID8	RNAscope PID9	
	Hyperintense Area (%)	DAPI Area (%)	DAPI + GFAP mRNA Area (%)
GB10	5.05	1.64	6.37
GB11	4.04	1.42	5.75
GB12	6.09	2.78	8.63

## Abbreviations

GB	Glioblastoma
MRI	Magnetic Resonance Imaging
EEG	Electroencephalography
mRNA	Messenger-Ribonucleic Acid
WHO	World Health Organization
LGG	Low-grade glioma
HGG	High-grade glioma
DRE	Drug-resistant epilepsy
MEG	Magnetoencephalography
PID	Post-inoculation day
M	MRI scan
E	EEG electrode implantation
R	RNAscope analysis
H	Histological analysis
H(●)	No histological analysis due to problems with histological processing
RWS	Fraction of rats with seizures per day
NCS	Total number of non-convulsive seizures
CS	Total number of convulsive seizures
DGi	Ipsilateral dentate gyrus
CA1i	Ipsilateral CA1
DGc	Contralateral dentate gyrus
Cp	Parietal cortex
GFAP	Glial Fibrillary Acidic Protein
SD	Standard deviation
DAPI	4′,6-diamidino-2-phenylindole
T	Tumor tissue
NT	Non-tumor tissue
I	Infiltrative spot
N	Necrotic spot
SEM	Standard error of the mean
SCID	Severe combined immunodeficiency
KCC2	Potassium chloride cotransporter 2
ATTC	American Type Culture Collection
DMEM	Dulbecco’s Modified Eagle Medium
FCS	Fetal calf serum
ECD	“Ethische Commissie Dierproeven”
AP	Anterioposterial
ML	Mediolateral
DV	Dorsoventral
PBS	Phosphate-buffered saline
C	Control
TR	Repetition time
TE	Echo time
TA	Acquisition time
NiDAQ	National instruments Data Acquisition
PFA	Paraformaldehyde
ISH	In situ hybridization
EtOH	Ethanol
NaCl	Sodium chloride
HRP	Horseradish peroxidase
TSA	Tyramide signal amplification
SPSS	Statistical Package for the Social Sciences
